# Once-weekly semaglutide doubles the five-year risk of nonarteritic anterior ischemic optic neuropathy in a Danish cohort of 424,152 persons with type 2 diabetes

**DOI:** 10.1186/s40942-024-00620-x

**Published:** 2024-12-18

**Authors:** Jakob Grauslund, Andreas Abou Taha, Laleh Dehghani Molander, Ryo Kawasaki, Sören Möller, Kurt Højlund, Lonny Stokholm

**Affiliations:** 1 Department of Ophthalmology, Odense University Hospital, J. B. Winsløws Vej 4, Odense C, DK-5000 Denmark; 2 Department of Clinical Research, University of Southern Denmark, Odense, Denmark; 3 Steno Diabetes Center Odense, Odense University Hospital, Odense, Denmark; 4 Division of Public Health, Department of Social Medicine, University of Osaka, Osaka, Japan; 5 Open Patient data Explorative Network, Odense University Hospital, Odense, Denmark

**Keywords:** Cohort study, Nonarteritic anterior ischemic optic neuropathy, Registry based, Semaglutide, Type 2 diabetes

## Abstract

**Background:**

Nonarteritic anterior ischemic optic neuropathy (NAION) is an untreatable condition often causing severe and irreversible visual loss in the affected eye. As it has recently been implied that the use of semaglutide associates with NAION, the aim of the present study was to evaluate this risk prospectively in all persons with type 2 diabetes (T2D) in Denmark.

**Methods:**

In a five-year longitudinal cohort study, we identified all persons with T2D in Denmark (*n* = 424,152) between 2018 and 2024. Patients were stratified according to exposure (*n* = 106,454) or non-exposure (*n* = 317,698) to once-weekly semaglutide, and incidence rates and hazard ratios (HR) of NAION were estimated in a multivariable Cox proportional hazard regression model.

**Results:**

At baseline, median age and hemoglobin A1c were 65 years and 50 mmol/mol, and 54·5% were male. During 1,915,120 person-years of observation, 218 persons developed NAION. Semaglutide exposure was associated with a higher incidence rate (0·228 vs. 0·093 per 1000 person-years, *p* < 0·001) and independently predicted a higher risk of upcoming NAION (HR 2·19, 95% confidence interval 1·54 − 3·12), even when multiple other factors were taken into account. Overall, 67 persons exposed to semaglutide developed NAION with a median time from first prescription to event of 22·2 months (interquartile range 10·2–37·8 months).

**Conclusions:**

During five years of observation of all persons with T2D in Denmark, use of once-weekly semaglutide independently more than doubled the risk of NAION. Given the irreversible nature of NAION, it is important to acknowledge this risk, and upcoming studies should aim to identify high-risk subgroups.

**Supplementary Information:**

The online version contains supplementary material available at 10.1186/s40942-024-00620-x.

## Background

Semaglutide is a glucagon-like peptide-1 receptor agonist that improves glycemic control and reduces cardiovascular outcomes in type 2 diabetes (T2D) by several mechanisms including enhanced β–cell response, postponed gastric emptying, inhibition of glucagon secretion, and weight-loss [1]. While semaglutide is in general considered safe, concerns have been raised that it may pose an increased risk of ocular disease, as evidenced by an increased risk of diabetic retinopathy worsening [2].

Nonarteritic anterior ischemic optic neuropathy (NAION) is another ocular disease that often presents with a sudden onset of painless monocular visual loss with altitudinal visual field defects. While the exact pathogenesis is unknown, it is likely caused by impaired perfusion of the optic nerve head. With an incidence of 11 per 100,000 person-years [3], it is the most common cause of optic neuropathy [4]. As it is non-treatable and often leads to severe and irreversible visual loss in the affected eye [4, 5], it is vital to identify potential risk factors that may predict NAION. Anatomic and systemic risk factors have traditionally included crowding of the optic nerve head, male gender, hypertension, hypercoagulability, and diabetes with end-organ damage [6], but recently Hathaway et al. proposed use of once-weekly semaglutide as a hereto unknown marker of risk. In 710 persons with T2D, semaglutide exposure associated with a hazard ratio (HR) of 4·28 (95% confidence interval [CI] 1·62 − 11·29) of upcoming NAION within three years [7]. This is an obvious concern given the almost 10-fold increase in the use of once-weekly semaglutide within the last five years [8].

The aim of the present study was to evaluate if exposure to once-weekly semaglutide predicted a higher absolute and relative five-year risk of NAION among all persons with T2D in Denmark.

## Methods

### Study cohort

In a national, registry-based prospective cohort study using multiple validated national registers, we identified all persons in Denmark who were alive and had T2D by December 1, 2018, or developed T2D no later than December 31, 2023. For identification, we combined data from the Danish National Patient Registry, including all diagnostic and treatment codes for in- and outpatient hospital care [9], and the Danish National Prescription Registry, containing information regarding redeemed prescriptions according to the Anatomical Therapeutical Chemicals (ATC) classification system [10]. T2D was defined by combining International Classification of Disease (ICD) version 10 codes [11] for T2D (E11*) and ACT-codes for insulin (A10A*) and non-insulin glucose-lowering medicine (A10B*) in accordance with definitions presented by Thykjaer et al. [12].

We included all persons with T2D above the age of 18 years free of NAION at the time of entry and excluded all persons that had previously used other forms of semaglutide (i.e. Rybelsus^®^ and Wegovy^®^) than once-weekly semaglutide.

### Exposures and outcome

In all persons with T2D, exposure was defined as redemption of at least one prescription of once-weekly semaglutide (Ozempic^®^, ATC: A10BJ06) as coded in the Danish National Prescription Registry between December 1, 2018, and December 31, 2023. The remaining persons with T2D were regarded as non-exposed. Index date was set as the day of the first redeemed prescription (exposed group) and December 1, 2018 (unexposed group). As we used time-varying exposure, patients exposed to once-weekly semaglutide participated as non-exposed prior to first redemption of semaglutide prescription.

The outcome was a diagnostic code of NAION (H470C) in the Danish National Patient Registry as registered between December 1, 2018, and June 30, 2024. In addition, we evaluated the overall number of persons with NAION (irrespective of T2D) between 1 January 2003 and 30 July 2024 to evaluate any overall trends over time.

### Covariates

Covariates were evaluated at the time of entry into the study.

Information regarding age, sex, and marital status was obtained from the Danish Civil Registration System, which was also used to link data from all registers by a unique personal identifier given to all inhabitants in Denmark at birth or immigration [13]. Duration of diabetes was calculated as the time between the first diagnostic code or redeemed prescription indicating T2D and the date of entry in the study. We used the Register of Laboratory Results for Research [14] to obtain measurements of hemoglobin A1c, plasma creatinine, albumin/creatinine ratio in urine, and estimated glomerular filtration rate (Supplementary Table 1). We used the registration in closest proximity to study inclusion within an allowed range of one year. Use of cholesterol (C10*) and blood pressure lowering medicine (C03*, C07*, C08*, and C09*) was determined in the Danish National Prescription Registry. Cardiovascular disease was defined in the Danish National Patient Registry as the first day of any of the diagnostic coding of Supplementary Table 2 [15]. Finally, we used the Danish Registry of Diabetic Retinopathy [16] to assess information of most recent level of diabetic retinopathy (as given by worse eye) according to the International Classification of Diabetic Retinopathy Disease Severity Scale [17], which is a five-step scale ranging from no diabetic retinopathy, through mild, moderate and severe nonproliferative diabetic retinopathy to end-stage proliferative diabetic retinopathy.

### Sensitivity analyses

To verify the robustness of the data, we performed three sensitivity analyses:

First, in order to account for potential unmeasured confounding by including an active comparator, we evaluated the risk of incident NAION in persons treated with sodium-glucose transport protein 2 (SGLT2) inhibitors (ATC: A10BK01 [*n* = 84,283], A10BK02 [*n* = 3048], A10BK03 [*n* = 85,892], A10BK04 [*n* = 96]) versus once-weekly semaglutide with or without redemption of prescriptions for SGLT2 inhibitors. In this analysis, patients were included from the redemption of the first prescription of a SGLT2 inhibitor or once-weekly semaglutide, whichever came first, until a month after the last prescript redemption or at the end of the study on June 30, 2024.

Second, we evaluated the risk of NAION among once-weekly semaglutide users in a model excluding persons with existing diabetic retinopathy to eliminate potential detection bias that may rise if patients who have been evaluated for diabetic retinopathy may be more prone to seek ophthalmic care.

Third, we excluded users of other glucagon-like peptide-1 receptor agonists to account for any potential class-effect of the drug.

### Statistical analyses

We present data as counts (with proportions) or medians (with interquartile ranges [IQR]). Differences between persons exposed and non-exposed in Table 1 were tested by the k-sample test for equality of medians (continuous data) and chi-square tests (categorical data).

**Table 1:** Baseline characteristics of persons with type 2 diabetes according to exposure of once-weekly semaglutide

	Overall	No semaglutide	Semaglutide	*p* value
Number of persons	424,152	317,698	106,454	
Sex				< 0·001
Male	231,360 (54·5%)	174,773 (55·0%)	56,587 (53·2%)	
Female	192,792 (45·5%)	142,925 (45·0%)	49,867 (46·8%)	
Age, years	65 (55–74)	68 (57–76)	58 (50–67)	< 0·001
Marital status				< 0·001
Never married	69,400 (16·4%)	48,461 (15·3%)	20,939 (19·7%)	
Married or living together	229,230 (54·1%)	169,708 (53·4%)	59,522 (55·9%)	
Divorced or widowed	125,471 (29·6%)	99,478 (31·3%)	25,993 (24·4%)	
Duration diabetes, years	3 (0–9)	2 (0–9)	4 (0–10)	< 0·001
HbA1c, mmol/mol	50 (44–59)	49 (43–57)	54 (47–65)	< 0·001
Plasma creatinine, mmol/mol	75·25 (63·67–90·67)	76·40 (64·50–92·50)	72·00 (61·50–85·50)	< 0·001
uACR, mmol/mol	15·00 (7·00–44·50)	15·00 (7·00–45·50)	14·00 (7·00–42·00)	< 0·001
eGFR, mmol/mol	84·00 (67·29–90·00)	82·00 (65·00–90·00)	89·00 (76·00–90·00)	< 0·001
Use of insulin				< 0·001
Yes	63,511 (15·0%)	40,323 (12·7%)	23,188 (21·8%)	
No	360,641 (85·0%)	277,375 (87·3%)	83,266 (78·2%)	
Use of non-insulin glucose lowering medicine				< 0·001
Yes	236,269 (55·7%)	169,356 (53·3%)	66,913 (62·9%)	
No	187,883 (44·3%)	148,342 (46·7%)	39,541 (37·1%)	
Cholesterol lowering medicine				< 0·001
Yes	276,896 (65·3%)	207,954 (65·5%)	68,942 (64·8%)	
No	147,256 (34·7%)	109,744 (34·5%)	37,512 (35·2%)	
Blood pressure lowering medicine				< 0·001
Yes	332,842 (78·5%)	251,744 (79·2%)	81,098 (76·2%)	
No	91,310 (21·5%)	65,954 (20·8%)	25,356 (23·8%)	
Cardiovascular disease				< 0·001
Yes	96,005 (22·6%)	79,474 (25·0%)	16,531 (15·5%)	
No	328,147 (77·4%)	238,224 (75·0%)	89,923 (84·5%)	
Level of diabetic retinopathy				< 0·001
No	126,232 (84·3%)	88,837 (85·1%)	37,395 (82·7%)	
Mild nonproliferative	14,729 (9·8%)	9,656 (9·2%)	5,073 (11·2%)	
Moderate nonproliferative	4,627 (3·1%)	2,987 (2·9%)	1,640 (3·6%)	
Severe nonproliferative	813 (0·5%)	488 (0·5%)	325 (0·7%)	
Proliferative	3,259 (2·2%)	2,470 (2·4%)	789 (1·7%)	

Data are presented as counts (with proportions) or medians (with interquartile ranges [IQR]). eGFR = estimated glomerular filtration rate. HbA1c = hemoglobin A1c. uACR = albumine/creatinine ratio in urine

In Table 2, we evaluated the number of first-time NAION events, person-years at risk, incidence rates, and we performed a crude and a multivariable Cox proportional hazard regression adjusted for sex, age, marital status, duration of diabetes, hemoglobin A1c, estimated glomerular filtration rate, history of cardiovascular disease, use of insulin, use of cholesterol lowering medicine, and use of blood pressure lowering medicine.

**Table 2:** Events, person-years at risk and HR for NAION according to exposure of once-weekly semaglutide

	Events of NAION	Person-years at risk (years)	Incidence rate (per 1000 person-years)	HR (95% CI)	
Semaglutide				Crude model	Mutivariable model*
Yes	67	294,395	0·228	2·57 (1·92 − 3·45)†	2·19 (1·54 − 3·12)†
No	151	1,620,725	0·093	Reference	Reference

CI = confidence interval. HR = hazard ratio. NAION = nonarteritic anterior ischemic optic neuropathy *Multivariable model adjusted for sex, age, marital status, duration of diabetes, hemoglobin A1c, estimated glomerular filtration rate, cardiovascular disease, use of insulin, use of cholesterol lowering medicine, and use of blood pressure lowering medicine. †Statistically significant

All persons were followed from the time of inclusion until the day of the first diagnostic coding of NAION, death, emigration or end of follow-up, whichever came first.

We used Stata 18.0 (StataCorp, College Station, Texas) for statistical analysis, and statistical significance was considered as p-values lower than 0·05 and 95% CIs that did not include 1.

## Results

We included 424,152 persons with T2D exposed (*n* = 106,454) or unexposed (*n* = 317,698) to once-weekly semaglutide. Among baseline characteristics presented in Tables 1 and 54·5% were male, the median age and duration of diabetes were 65 and 3 years, hemoglobin A1c was 50 mmol/mol, estimated glomerular filtration rate was 84·00 mmol/mol, and 15·0% were using insulin.

Persons exposed to once-weekly semaglutide were more likely to be married or living with a partner, had a longer duration of diabetes, higher values of hemoglobin A1c and estimated glomerular filtration rate, and were more likely to use insulin and non-insulin glucose-lowering medicine, and to have diabetic retinopathy. They were also younger and less likely to be male, to use cholesterol lowering and blood pressure lowering medicine, to have cardiovascular disease, and they also had lower plasma creatinine and albumin/creatinine ratio in urine.

**Fig. 1:**
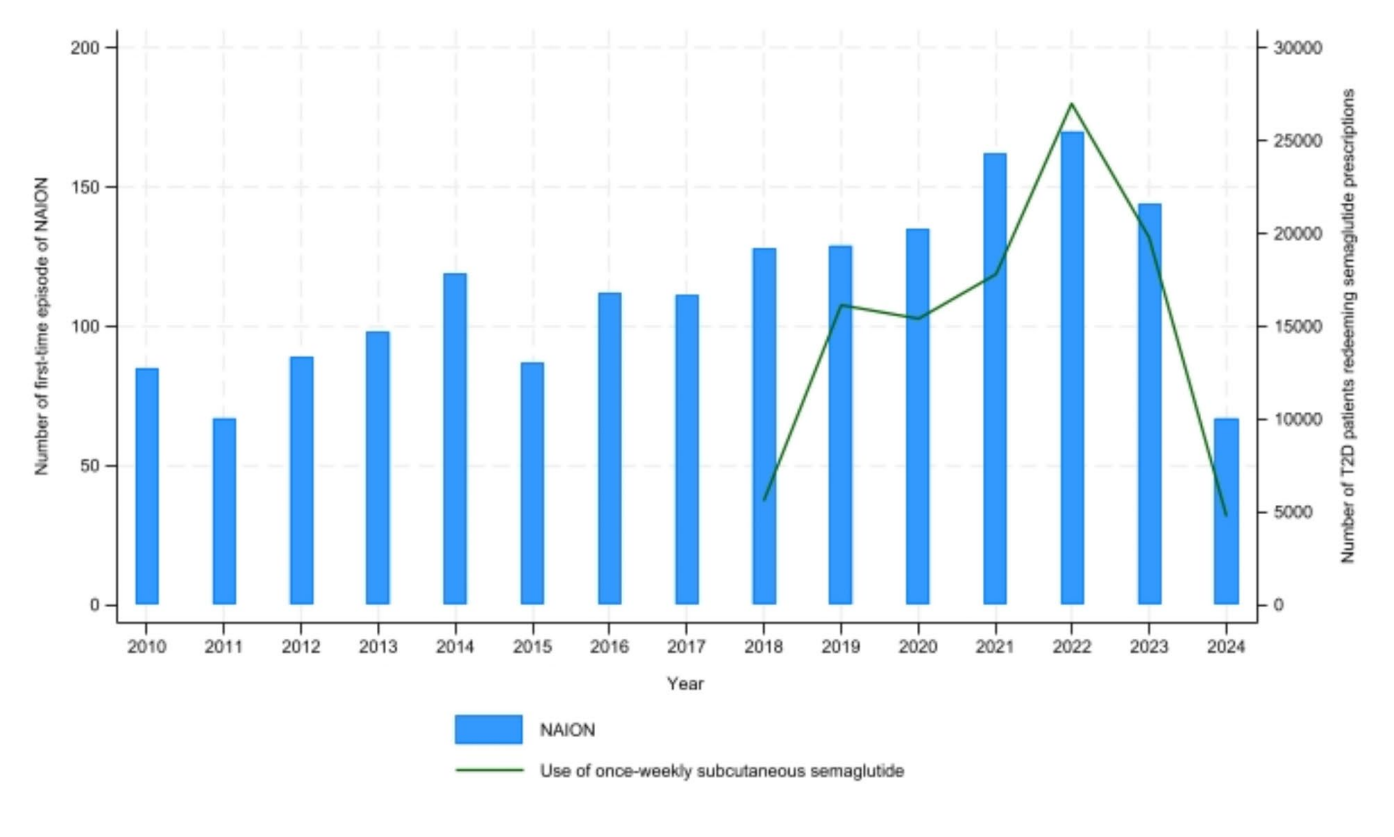
Number of first-time episodes of nonarteritic anterior ischemic optic neuropathy (NAION) and persons with type 2 diabetes (T2D) redeeming at least one prescription of once-weekly semaglutide (Ozempic^®^) between January 1, 2010, and June 30, 2024

Between 2003 and 2023, the five years with the highest numbers of first-time NAION events were 2019–2023, corresponding to the first five full years once-weekly semaglutide has been available on the marked (Fig. 1). The annual number of first-time NAION episodes was 67·6 in 2003–2018 and 148·0 in 2019–2023. Likewise, the rate of prevalent T2D among patients with newly-diagnosed NAION raised from 4·0% in 2003–2018 to 24·7% in 2019–2023.

Among 1,915,120 person-years of observation in persons with T2D, 218 developed NAION, corresponding to 0·114 per 1000 person-years. Exposure to once-weekly semaglutide was followed by 67 events of NAION during 294,395 years of observation as compared to 151 events during 1,620,725 years of observation for non-exposed, reflecting a higher incidence rate (0·228 vs. 0·093 per 1000 person-years, *p* < 0·001), as presented in Table 2.

In the crude Cox proportional hazard regression model, HR of NAION among once-weekly semaglutide users was 2·57, 95% CI 1·92 − 3·45. In the multivariable Cox proportional hazard regression model adjusted for sex, age, marital status, duration of diabetes, hemoglobin A1c, estimated glomerular filtration rate, cardiovascular disease, use of insulin, use of cholesterol lowering medicine, and use of blood pressure lowering medicine, exposure to once-weekly semaglutide also was independently associated with a higher risk of upcoming NAION (HR 2·19, 95% CI 1·54 − 3·12).

**Fig. 2:**
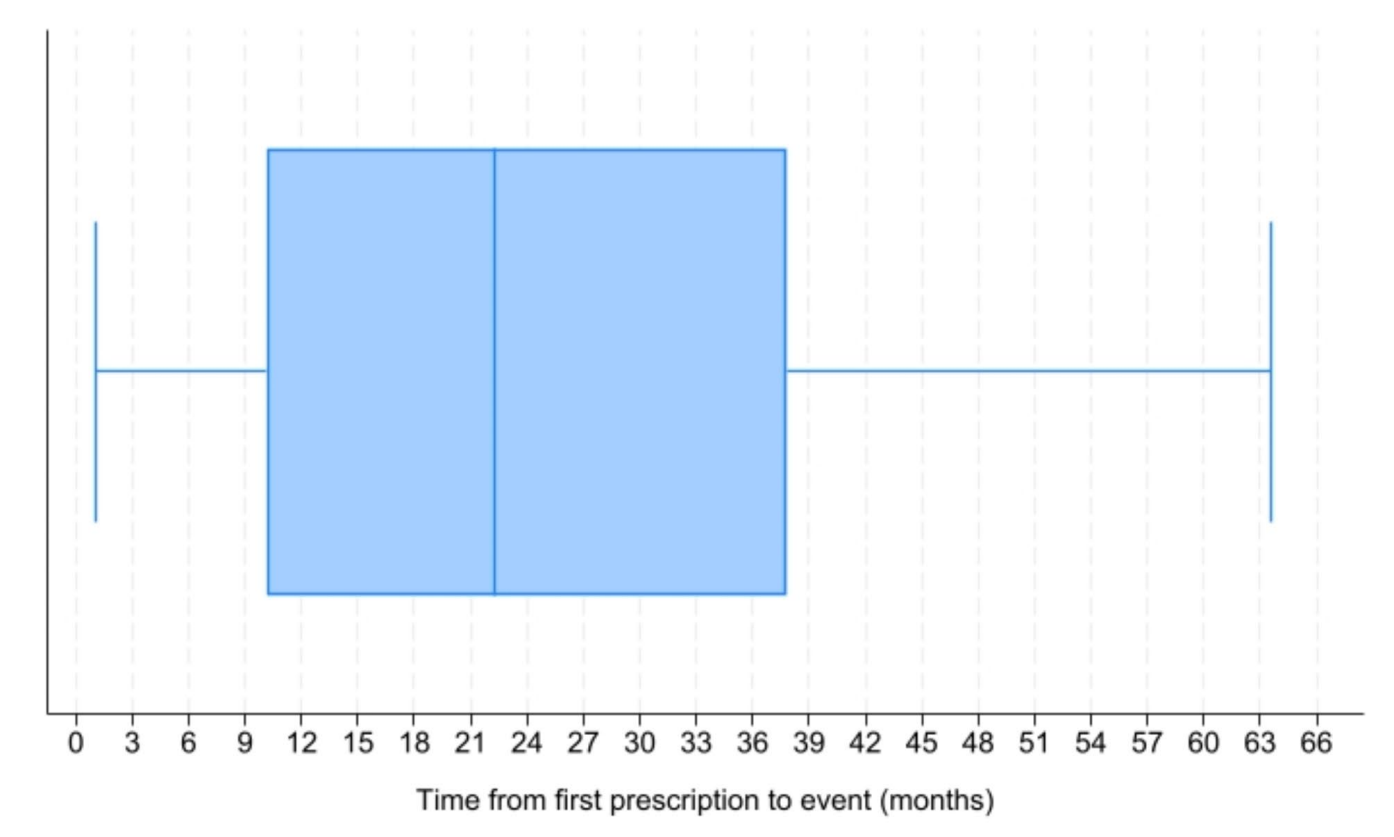
Time from redemption of first prescription of once-weekly semaglutide (Ozempic^®^) to first event of nonarteritic anterior ischemic optic neuropathy (NAION) in the 67 persons with type 2 diabetes patients, who were exposed to semaglutide and developed at least one event of NAION in 2018–2024. Box and whisker plot includes median time, interquartile range and range

For the 67 patients exposed to once-weekly semaglutide that subsequently developed NAION, median time from redemption of first prescription to NAION was 22·2 months (IQR 10·2–37·8 months) with no lower or upper time window between first exposure and event (Fig. 2).

In the first sensitivity analysis comparing the risk of incident NAION in persons with T2D treated with SGLT2 inhibitors alone (*n* = 40,206, reference), once-weekly semaglutide alone (*n* = 71,658), and once-weekly semaglutide and SGLT2 inhibitors combined (*n* = 45,401), we observed 6 events in 82,877 person-years, 36 events in 178,868 person-years, and 31 events in 119,106 person-years, respectively, corresponding to adjusted HR of 2·42 (95% CI 0·97 − 6·06) and 2·62 (95% CI 1·02–6·74) for once-weekly semaglutide without and with SGLT2 inhibitors in a multivariable model adjusted for sex, age, marital status, duration of diabetes, hemoglobin A1c, estimated glomerular filtration rate, cardiovascular disease, use of insulin, use of cholesterol-lowering medicine, and use of blood pressure lowering medicine.

When excluding patients who had been diagnosed with diabetic retinopathy in the second sensitivity study, use of once-weekly semaglutide was still independently associated with a higher risk to develop NAION (adjusted HR 2·26, 95% CI 1·57 − 3·27) in a multivariable model adjusted as in the first sensitivity analysis.

In the third sensitivity analysis, excluding patients using other types of glucagon-like peptide-1 receptor agonists, the use of once-weekly semaglutide still independently predicted a higher risk of upcoming NAION (multivariable adjusted HR 2·34, 95% CI 1·54 − 3·57), compared to non-semaglutide users in a multivariable model adjusted as in the first sensitivity analysis.

## Discussion

In the present study, we are the first to demonstrate in an entire national cohort of 424,152 persons with T2D that the use of once-weekly semaglutide independently predicted a 2·19 fold increased hazard to develop NAION, an untreatable disease most frequently causing severe and irreversible visual loss in the affected eye. In fact, after the introduction of once-weekly semaglutide in Denmark in November 2018, the annual number of first-time NAION episodes reached an all-time high for the years 2019–2023. Likewise, at the same time the rate of T2D in newly-diagnosed NAION raised from one in 20 to one in four.

Our study supports recent findings from Hathaway et al. that were the first to indicate an increased risk of NAION in persons exposed to once-weekly semaglutide [7]. Using data from a retrospective matched cohort of a centralized register of patients evaluated by neuro-ophthalmologists at Massachusetts Eye and Ear, Boston, MA, USA, a HR of 4·28 (95% CI 1·62 − 11·29) was found in 710 persons with T2D of whom 194 had been exposed to semaglutide. In comparison, we studied 424,152 persons in a national cohort. Persons from our cohort were in general older (65 years vs. 59 years), more likely to be male (54·5% vs. 48·0%), but less likely to have cardiovascular disease (22·6% vs. 43·8%). Likewise, Hathaway et al. reported of a cumulative 36-month incidence of 8·9% among semaglutide users with T2D [7] in comparison to an annual incidence of 0·228 per 1,000 person-years in our national cohort. In particular the latter is likely to reflect the difference between a selected population at a tertiary referral center and a real-world national cohort, but at the same time our findings confirm that the elevated risk associated with once-weekly semaglutide is a general phenomenon in T2D and not restricted to selected high-risk populations.

The risk of NAION has traditionally been attributed to anatomical crowding of the optic disc (“disc at risk”) as well as systemic cardiovascular risk factors like hypertension, dyslipidemia, and T2D [18]. Based on case-reports, it has previously been speculated that use of drugs like phosphodiesterase-5 inhibitors may also increase the risk of NAION [19]. Given the rarity of the disease, such cause and effect relationships are difficult to prove in clinical registration studies, but rather rely on subsequent large-scale, real-world studies like ours.

We were not able to find any high-risk window between exposure and outcome in persons with T2D diabetes exposed to once-weekly semaglutide that subsequently developed NAION. On the contrary, median time to event was 22·2 months, and the onset of NAION was evenly distributed within the entire five-year observation period. In contrast, Hathaway et al. found the highest risk within the first year following prescription of semaglutide.

Even though we have demonstrated a higher risk of NAION in persons with T2D exposed to once-weekly semaglutide, it is important to keep in mind that use of semaglutide comes with substantial advantages for patients as given by the improved glycemic control, reduction in risk of cardiovascular disease as well as the beneficial effects of weight loss [1]. As such, the observed incidence rate of NAION of 0·228 per 1000 person-years for persons with T2D exposed with once-weekly semaglutide may not discourage semaglutide treatment but needs to be acknowledged as a potential risk.

As the pathogenic pathway of NAION is insufficiently understood, it is difficult to speculate as to how semaglutide may lead to elevated risk. Expression of glucagon-like peptide-1 receptor agonist receptors have been identified in the optic nerve [20], but it is unknown whether continuous stimulation of these with specific glucagon-like peptide-1 receptor agonists may alter vascular perfusion of the optic nerve head. In SUSTAIN-6, use of once-weekly semaglutide led to an increased risk of diabetic retinopathy worsening (HR 1·76, 95% CI 1·11 − 2·76) [1], and it has been speculated that this can likely be attributed to early worsening given the rapid improvement in glycemic control in the semaglutide arm [21]. While persons exposed to semaglutide in our study also had impaired glycemic control (hemoglobin A1c 54 vs. 49 mmol/mol in non-exposed group), our findings of an increased risk of NAION were still statistically significant after adjustment for glycemic control, indicating a likely different mode of action as in diabetic retinopathy.

While our study is strengthened by the longitudinal design with five-year data from an entire national cohort of persons with T2D based on validated, national registers in a tax-funded health care society, limitations are also important to acknowledge. First, while we were able to adjust for multiple potential confounders, we did not have access to smoking, blood pressure, or body mass index. Second, as national cohort studies are only conclusive given a sufficient amount of exposure as well as adequate observational time for development of outcome, we are currently not able to expand the findings to oral semaglutide (Rybelsus^®^) in T2D (insufficient exposure) or once-weekly semaglutide (Wegovy^®^) in obesity (lacking time for development of outcome). Third, as this was a registry-based study, we did not have access to ophthalmic examinations but only the conclusive diagnostic code (NAION). Fourth, we were only able to evaluate redeemed prescriptions but could not assess, if the patients actually took the medicine as prescribed. Fifth, data on race or ethnicity could not be included, as this is not available in the Danish registers. Sixth, while exposure of once-weekly semaglutide for some patients were temporarily linked with incident NAION, our study cannot claim a causal relationship, as we do not know the underlying pathogenic mechanisms. Seventh, we were not able to examine the importance of duration of exposure in the analyses, as the relative rarity of NAION would decrease the statistical power of the study considerably and also violate the General Data Protection Regulations that individualized patients should not be identifiable in register-based studies.

In conclusion, we have demonstrated in a five-year national cohort study that use of once-weekly semaglutide more than doubles the risk of NAION, even when multiple other factors have been taken into account. As optic neuropathies are untreatable and irreversible, particular care should be given to avoid onset. For upcoming studies, it would be important to identify any potential high-risk subgroups as well as assess whether the elevated risk of NAION is a drug class effect or a specific finding for subcutaneously administered semaglutide.

## Electronic supplementary material

Below is the link to the electronic supplementary material.

Supplementary Material 1

## Data Availability

The data dictionary, the statistical analysis plan, and analytic coding can be made available from the corresponding author upon reasonable request. According to Danish law, the dataset or individual participant data cannot be shared.

## Abbreviations

ATC: Anatomical Therapeutical Chemicals
CI: Confidence interval
HR: Hazard ratio
ICD: International Classification of Disease
IQR: Interquartile ranges
NAION: Nonarteritic anterior ischemic optic neuropathy
SGLT2: Sodium-glucose transport protein 2
STROBE: Strengthening the Reporting of Observational Studies in Epidemiology
T2D: Type 2 diabetes
