# Supplementary material for: Once-weekly semaglutide doubles the five-year risk of nonarteritic anterior ischemic optic neuropathy in a Danish cohort of 424,152 persons with type 2 diabetes
Source: Int J Retina Vitreous. 2024 Dec 18;10:97. doi: 10.1186/s40942-024-00620-x (PMC11657653; doi:10.1186/s40942-024-00620-x)
Supplement: Supplementary file 1 — Supplementary Material 1 [file 40942_2024_620_MOESM1_ESM.docx]

**Supplementary Table 1**

Codebook of diagnostic codes used to extract blood-sampling measurements from the Register of Laboratory Results for Research.

| **Blood measurement** | **NPU/DNK** |
| --- | --- |
| Plasma creatinine* | NPU04998, NPU01807, NPU09101, NPU18016, NPU17559, NPU18105, NPU08615, NPU26918 |
| eGFR* | DNK35131, DNK35301, DNK35302, DNK35304, NPU19597 |
| uACR* | NPU19661, NPU28842 |
| HbA1c* | NPU377226, NPU27300 |

eGFR=estimated glomerular filtration rate. HbA1c=hemoglobin A1c. NPU/DNK=Nomenclature for properties and units according to Danish standards. uACR=albumine/creatinine ratio in urine. *Missing values of plasma creatinine, eGFR, uACR, and HbA1c were 10%, 11%, 55%, and 18%, respectively.

**Supplementary Table 2**

Codebook of diagnostic codes used to define cardiovascular disease in the Danish National Patient Registry.

| **Cardiovascular disease** | **ICD-10** |
| --- | --- |
| Myocardial infarction |  |
| First-time and recurrent myocardial infarction | I21*- I23*, I240*- I241*, I248, I249* |
| Heart failure |  |
| First-time heart failure + re-admission for heart failure | I110, I130, I132, I420, I426 - I427, I429, I500*- I501*, I509* |
| Ischemic stroke |  |
| Stroke, ischemic/Transient ischemic attack | I63*-I64*, G458, G459 |
| Intracranial hemorrhage |  |
| Stroke, cerebrovascular bleeding | I60*-I62* |
| Peripheral arterial disease |  |
| Peripheral arterial disease | I702, I702A, I739A, I739C |
| Other arterial thromboembolic occlusion | I74* |

ICD-10=International Classification of Disease, version 10. *Includes sub-codes.
